# Development and validation of a novel nomogram predicting axillary lymph node metastasis among breast cancer patients in Egypt

**DOI:** 10.1038/s41598-026-37354-9

**Published:** 2026-02-17

**Authors:** Horeya Mohamed Ismail, Mostafa Ahmed Arafa, Amr Abdel Aziz Elsaid, Mohamed Mostafa Tahoun

**Affiliations:** 1 Department of Epidemiology, High Institute of Public Health, Alexandria University, 65 El-Horeya Road, Al Ibrahimeyah Qebli WA Al Hadrah Bahri, Bab Shar’, 5422031 Alexandria Governorate Egypt; 2 Department of Clinical Oncology, Faculty of Medicine, Alexandria University, Alexandria, Egypt

**Keywords:** Axillary lymph node dissection (ALND), Nomogram, Decision curve analysis, Personalized axillary management

## Abstract

Axillary lymph node dissection (ALND) remains widely used in the management of breast cancer in Egypt despite its association with significant morbidity. A reliable tool for predicting axillary lymph node metastasis (ALNM) could help reduce unnecessary surgical interventions. This study aimed to develop and validate a predictive nomogram to estimate the risk of ALNM in patients with breast cancer. We conducted a retrospective study of 1246 women with invasive breast cancer (stages I–III) treated at a tertiary center in Alexandria, Egypt (2018–2024). A perioperative, pathology-assisted model was developed as the primary tool, and a secondary, preoperative-only model was evaluated as a sensitivity analysis. A nomogram was constructed using multivariable logistic regression to predict ALNM, and model performance was assessed using the area under the ROC curve (AUC), calibration plots, and decision curve analysis (DCA). Five independent predictors of ALNM were identified. The primary model, which was developed using all clinically important and statistically significant variables, demonstrated excellent discrimination (AUC = 0.917) with good calibration. A sensitivity analysis yielded a preoperative-only model with highly comparable performance (AUC = 0.898). Decision curve analysis supported the use of a 20% risk threshold, at which the model achieved 93.9% sensitivity, 59.5% specificity, and a negative predictive value of 87.7%. It also confirmed a superior clinical net benefit compared to axillary sonography alone or default strategies. Our nomogram offers a practical, noninvasive tool to guide personalized axillary management in Egyptian patients with breast cancer, supporting risk-stratified decisions that may safely reduce unnecessary axillary surgery and align with global de-escalation principles.

**Supplementary Information:**

The online version contains supplementary material available at 10.1038/s41598-026-37354-9.

## Introduction

The management of breast cancer (BC) involves a combination of treatment modalities, such as surgery, radiation therapy, chemotherapy, and hormonal therapy, tailored to the tumor’s stage and clinical features^1^. Among these treatment options, surgical management plays a crucial role in both the diagnosis and treatment of BC. It represents the primary approach for controlling local disease; however, the potential for cancer cells to spread through the lymphatic vessels to the axillary lymph nodes emphasizes the critical importance of assessing the status of axillary lymph nodes as a prognostic factor influencing treatment decisions and patient outcomes^2^.

Axillary lymph node metastasis (ALNM) refers to the spread of cancer from the primary breast tumor to the axillary lymph nodes. For many years, the standard procedure to assess axillary lymph node involvement has been axillary lymph node dissection (ALND), which involves the surgical removal of a significant number of lymph nodes from the axilla. Histopathological evaluation of excised nodes provides essential information for accurate staging and guiding subsequent treatment strategies^3^.

Indeed, ALND has shown benefits in controlling regional nodal disease and has the potential to improve overall survival^4^. However, it is important to acknowledge that ALND is a highly invasive surgical approach, which leads to notable side effects and considerable morbidity^5^. Frequently reported complications include surgical site infections, lymphedema of the arm, lymphangitis, numbness, shoulder dysfunction, and limitations in arm mobility, all of which can severely impact a patient’s quality of life^6^.

Recent advancements in BC screening and diagnostic technologies have facilitated earlier tumor detection and reduced nodal involvement rates, especially in patients with early stage tumors. Evidence suggests that > 75% of axillary lymph nodes excised during ALND in such patients are histologically negative, highlighting significant overtreatment in many cases^7,8^.

Sentinel lymph node biopsy (SLNB) has revolutionized the surgical management of axillary lymph nodes by introducing a less invasive approach than extensive ALND, particularly for patients with early stage breast cancer. This has become the standard of care for clinically node-negative cases. However, SLNB remains an invasive procedure that carries some complications similar to those of ALND, as well as the potential for false-negative results, which may adversely impact treatment planning and patient outcomes^9,10^. These limitations highlight a significant gap in current clinical practice, as there is no reliable, non-invasive, and accurate method for evaluating axillary lymph node involvement before surgical decision-making. Commonly used imaging modalities, such as ultrasound, often face challenges in detecting small or early-stage lymph node metastases and have reported false-negative rates as high as 22.9%^11^.

To address this gap, there is a compelling need for a novel, non-invasive predictive tool capable of accurately assessing the risk of axillary lymph node involvement preoperatively. Such a tool would complement existing surgical strategies by providing a preliminary risk evaluation that would enable clinicians and surgeons to make more informed decisions and avoid unnecessary invasive procedures whenever possible, particularly in patients with low-risk profiles.

Nomograms, which graphically represent predictive models, offer a promising solution by translating complex statistical analyses into intuitive tools for personalized risk prediction. A validated nomogram could enable clinicians and surgeons to stratify patients based on their risk of nodal involvement, thereby facilitating tailored treatment decisions, avoiding unnecessary ALND, and minimizing associated morbidity. By incorporating decision curve analysis, the validity, practical value, clinical applicability, and utility of the developed nomogram in a real-world setting can be realized, as it will contribute valuable evidence to decision support, ensuring that unnecessary axillary dissections are avoided when they may not be beneficial for certain subgroups of BC patients^12,13^.

This study aimed to develop and validate a predictive nomogram to estimate the preoperative risk of axillary lymph node metastasis in patients with breast cancer. By offering a personalized risk assessment tool, the nomogram may provide a clinical decision-support tool regarding appropriate axillary management and help avoid unnecessary interventions, thereby reducing treatment-related morbidity and improving quality of life. Its clinical utility will be further evaluated through decision curve analysis to support its integration into routine BC care.

## Methods

### Study design and target population

This retrospective study was conducted at a specialized tertiary care center in Alexandria, Egypt. It included women diagnosed with invasive breast cancer (TNM stage I–III) who underwent ALND and/or SLNB between 2018 and 2024. Patients were excluded if they had recurrent disease, distant metastasis, or a history of neoadjuvant therapy. Eligible participants met the following criteria: female sex, single primary tumor, complete clinical and pathological data, available preoperative radiological imaging, and pathologically confirmed lymph node status. Patients were stratified into ALN-positive and ALN-negative groups based on pathological findings.

### Ethics approval and consent to participate

The study adhered to international research ethics guidelines and followed the principles of the 1964 Declaration of Helsinki and its subsequent amendments. The study was approved by the Ethics Committee of the High Institute of Public Health at Alexandria University, Egypt (IRB number: 00013692; Ethical Approval number: AU0923926267; issued on 26/09/2023). The study involved a retrospective analysis of anonymized electronic health records with no direct patient contact, and individual informed consent was therefore exempted by the Institutional Review Board.

### Data collection and management

Eligible patient records were systematically reviewed, and data were extracted using a standardized case report form to ensure consistency and completeness. The following information was collected:

- *Patient characteristics*: age and body mass index.
- *Medical and reproductive history*: menopausal status, comorbidities, surgical history, and family history of breast cancer.
- *Clinicopathological characteristics*: tumor size, histological type, histological grade, receptor status (ER, PR, HER2), lymphovascular invasion (LVI), and molecular subtype.
- *Preoperative radiological findings*: mammography and axillary sonography, skin involvement, and nipple involvement.
- *Surgical and pathological details*: type of breast and axillary surgery, number of dissected and positive lymph nodes, Perineural Invasion (PNI), extranodal fat infiltration, and pathological TNM stage.

Variables were selected based on a systematic review of established predictors of axillary metastasis, biological plausibility, and availability in routine clinical practice. We prioritized factors that would be available at the time of surgical decision-making to enhance practical utility.

The ALNM status was determined from the final histopathological examination. For patients who underwent ALND, nodal status was based on complete axillary dissection specimens, whereas for those who underwent SLNB, nodal status was based on sentinel lymph node pathology. In cases with SLNB followed by completion of ALND, the combined pathology results were used.

Tumor laterality and location were recorded as the right, left, central region, axillary tail, or specific quadrant (UOQ, LOQ, UIQ, LIQ). Histologic type was classified based on fine-needle aspiration or core biopsy results as invasive ductal carcinoma (IDC), invasive lobular carcinoma (ILC), or less common subtypes, such as mucinous carcinoma or medullary carcinoma. Hormone receptor and HER2 status were documented as positive or negative according to institutional immunohistochemistry protocols, and molecular subtype was determined as Luminal A (HR+/HER2–), Luminal B (HR+/HER2+), HER2-enriched (HR–/HER2+), or triple-negative (HR–/HER2–). Tumor size followed the AJCC categories (T1 ≤ 2 cm, T2 2–5 cm, and T3–T4)^14^. Ki-67 proliferation index was categorized as low (< 14%), intermediate (14–19%), or high (≥ 20%)^15^. Skin involvement, nipple involvement, and LVI were recorded as binary variables.

The LVI status was obtained from pathology reports. In some cases, LVI was reported from preoperative core or incision biopsy specimens, whereas in most patients, it was documented in postoperative surgical pathology reports. Because biopsy samples represent only a limited portion of the tumor and surrounding tissue, LVI may be underdetected preoperatively; therefore, postoperative histopathology provides a more reliable source of LVI information in this cohort.

Axillary lymph node ultrasonography findings were classified as reactive for benign-looking LN or with pathological features, including cortical thickening, loss of fatty hilum, abnormal shape, irregular margins, and abnormal vascularity^16^.

### Statistical analysis

Descriptive statistics were generated to summarize the study population. Categorical variables are presented as frequencies and percentages, whereas continuous variables are assessed for normality and summarized using means and standard deviations or medians and interquartile ranges, as appropriate. Comparisons between the ALN-positive and ALN-negative groups were performed using the chi-square test or Fisher’s exact test.

Univariate logistic regression analyses were performed to identify potential predictors of axillary lymph node metastasis. The odds ratios (OR) with 95% confidence intervals (CI) and P-values are presented. Variables with clinical relevance or statistical significance in the univariate analysis were subsequently entered into a multivariate logistic regression model. A predictive nomogram was constructed based on the final multivariate model.

Multivariable logistic regression analysis was performed to identify the independent risk factors associated with ALN status among the variables showing statistical significance in the univariate analyses (*P* < 0.0.05) using the enter method. The primary model involved all statistically significant and clinically important variables. Model performance discrimination was evaluated using the area under the receiver operating characteristic curve (AUC/ROC). and the Hosmer–Lemeshow goodness-of-fit test.

A nomogram for ALNM was developed from the multivariable logistic regression model (*P* < 0.05). Model performance was assessed using the concordance index (C-index) and calibration plots generated through bootstrap resampling. The C-index is a numerical measure that quantifies discriminative ability, whereas calibration plots are graphic evaluations of the predictive ability that compare observed outcomes with nomogram-predicted probabilities to assess calibration. Receiver operating characteristic (ROC) curves were also constructed, and the area under the curve (AUC) was calculated to further evaluate model discrimination.

To assess the robustness and preoperative applicability of the model, a sensitivity analysis was conducted in which a preoperative-only model was refitted after excluding postoperative variables (including LVI). This analysis allowed the evaluation of whether reliable discrimination could be achieved using preoperative information alone. The performance of the sensitivity model was assessed using the same discrimination and calibration metrics as those used for the primary model.

The clinical utility of the nomogram was evaluated using decision curve analysis (DCA), which quantifies the net benefit and net reduction in unnecessary axillary management decisions across a range of threshold probabilities.

In the decision curve analysis, the thresholds represent patients classified as high risk of lymph node involvement, while the choice of surgical intervention (SLNB or ALND) at a given threshold is context-dependent and guided by prevailing clinical practice guidelines rather than determined by the model itself.

Statistical analyses were performed using SPSS version 26.0 (IBM, Armonk, NY, USA) for descriptive statistics as well as univariate and multivariable analyses. The nomogram was constructed in R version 4.0.3 using the “rms” package.

## Results

### Patient cohort

**Fig. 1:**
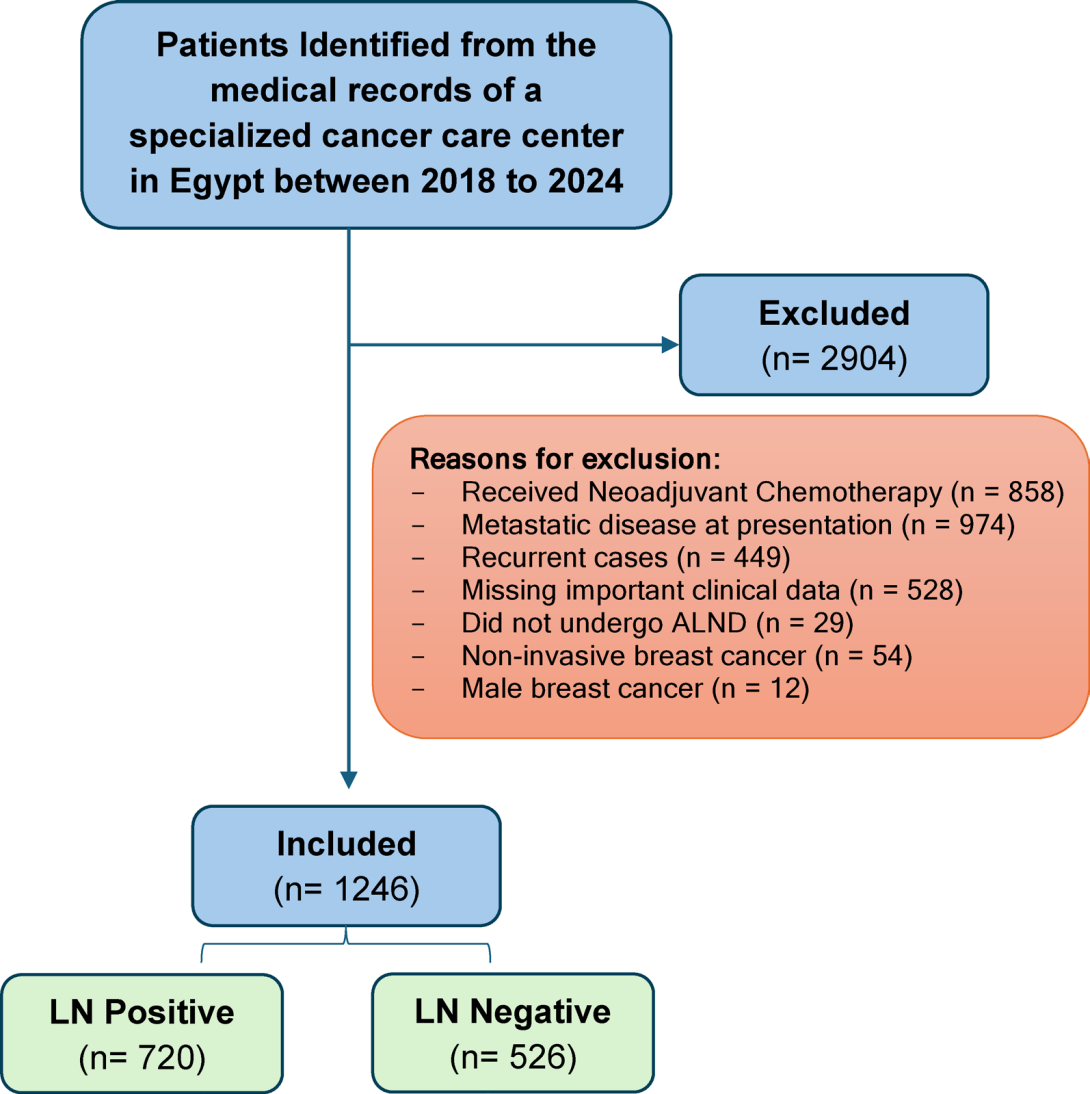
Flow chart of patient selection.

A total of 4150 breast cancer cases were identified from the institutional database between 2018 and 2024. After applying the predefined eligibility criteria, 1,246 women with invasive breast cancer were included in the final analysis (Fig. 1). Among them, 526 patients (42.2%) had no axillary lymph node (ALN) metastasis, whereas 720 (57.8%) demonstrated pathologically confirmed ALN involvement. Regarding breast surgery, 604 patients (48.5%) underwent mastectomy, and 642 (51.5%) underwent breast-conserving surgery. In terms of axillary management, 932 patients (74.8%) underwent ALND, 277 (22.2%) underwent SLNB, and 37 (3.0%) required SLNB followed by ALND. Overall, 501 patients (40.2%) had fewer than 10 lymph nodes assessed, whereas 745 (59.8%) had more than 10 lymph nodes dissected. Based on pathological staging, 17.2% were Stage I, 50.5% were Stage II, and 32.3% were Stage III. Perineural invasion was observed in 121 patients (9.7%).

The patient characteristics are detailed in Table 1. The cohort ranged in age from 25 to 87 years, with a mean age of 55.2 years (SD ± 12.37); 760 patients (61.0%) were above 50 years. Obesity was common (827 patients; 66.4%), and among obese women, 57.9% had ALN metastasis. More than half of the patients were postmenopausal (56.9%). Comorbidities were present in 47.4% of patients, previous surgical history in 52.2%, and family history of breast cancer in 52.2%. None of these variables differed significantly between the ALN-positive and ALN-negative groups.

**Table 1:** Distribution of patients characteristics by lymph node status.

Characteristics	Negative(N = 526) N (%)	Positive (N = 720) N (%)	Total (N = 1246) N (%)	*P*-value
Age of diagnosis (years)				
Minimum- maximum Mean (SD)			25—87 55.2 (12.37)	
20—30	5 (41.7)	7 (58.3)	12 (1.0)	0.717
31—40	62 (44.9)	76 (55.1)	138 (11.0)	
41—50	148(44.0)	188 (56.0)	336 (27.0)	
> 50	311 (40.9)	449 (59.1)	760 (61.0)	
BMI				0.530
Underweight	1 (33.3)	2 (66.7)	3 (0.2)	
Healthy weigh	51 (48.6)	54 (51.4)	105 (8.4)	
Overweight	126 (40.5)	185 (59.5)	311 (25.0)	
Obesity	348 (42.1)	479 (57.9)	827 (66.4)	
Menopausal status				0.504
Premenopausal	221 (43.5)	287 (56.5)	508 (40.8)	
Postmenopausal	294 (41.5)	415 (58.5)	709 (56.9)	
Perimenopause	4 (26.7)	11 (73.3)	15 (1.2)	
Amenorrheic/surgical conditions	7 (50.0)	7 (50.0)	14 (1.1)	
Comorbidities				0.075
Absent	292 (44.6)	363 (55.4)	655 (52.6)	
Present	234 (39.6)	357 (60.4)	591 (47.4)	
Surgical history				0.199
Absent	240 (40.3)	355 (59.7)	595 (47.8)	
Present	286 (43.9)	365 (56.1)	651 (52.2)	
Family history of BC				0.285
Absent	360 (41.2)	513 (58.8)	873 (70.1)	
Present	166 (44.5)	207 (55.5)	373 (29.9)	

*BMI* body mass index.

### Clinicopathologic tumor characteristics by lymph node status

Univariate analysis of clinicopathological variables identified several factors significantly associated with axillary lymph node (ALN) metastasis (Table 2). Tumor location within the breast showed a strong association with nodal involvement (*P* < 0.001), with lesions in the axillary tail and overlapped- or multiple-site tumors demonstrating particularly high metastatic rates. A higher histologic grade was also associated with increased nodal positivity (*P* = 0.031). Multifocality showed a progressive increase in ALN metastasis from single tumors to bifocal and multifocal tumors (*P* < 0.001). Tumor size demonstrated a clear stepwise relationship, with nodal involvement rising markedly from T1 to T3–T4 lesions (*P* < 0.001). Molecular subtype was significantly associated with ALN status (*P* = 0.031), with HER2-enriched and Luminal B tumors exhibiting higher metastatic proportions, whereas triple-negative cancers demonstrated comparatively lower nodal positivity. Skin and nipple involvement was strongly linked to ALN metastasis (*P* = 0.002 and *P* = 0.001, respectively). Preoperative axillary ultrasonography findings showed one of the most pronounced associations: 90.0% of lymph nodes with pathological sonographic features were metastatic, compared to only 20.7% of reactive nodes (*P* < 0.001). Likewise, lymphovascular invasion (LVI) demonstrated a strong relationship with nodal metastasis, with 74.5% of LVI-positive tumors showing ALN involvement versus 34.2% of LVI-negative tumors (*P* < 0.001). All variables that were statistically significant in the univariate analysis were subsequently included in the multivariate logistic regression model to identify the independent predictors of ALN metastasis.

**Table 2:** Distribution of tumor clinicopathological characteristics by axillary lymph node status.

Characteristics	Negative(N = 526) N (%)	Positive (N = 720) N (%)	Total (N = 1246) N (%)	*P*-value
Laterality				0.262
Right	249 (40.6)	364 (59.4)	613 (49.2)	
Left	277 (43.8)	356 (56.2)	633 (50.8)	
Quadrant				< 0.001*
Central region	113 (51.6)	106 (48.4)	219 (17.6)	
Axillary tail	23 (13.2)	151 (86.8)	174 (14.0)	
Multiple sites	19 (24.4)	59 (75.6)	78 (6.3)	
UOQ	224 (42.5)	303 (57.5)	527 (42.3)	
LOQ	37 (52.1)	34 (47.9)	71 (5.7)	
UIQ	63 (68.5)	29 (31.5)	92 (7.4)	
LIQ	47 (55.3)	38 (44.7)	85 (6.8)	
Histologic type				0.108
IDC	427 (42.1)	588 (57.9)	1015 (81.5)	
ILC	73 (39.5)	112 (60.5)	185 (14.8)	
Others	26 (56.5)	20 (43.5)	46 (3.7)	
Histologic grade				0.031*
1	23 (62.2)	14 (37.8)	37 (3.0)	
2	416 (42.1)	571 (57.9)	987 (79.2)	
3	87 (39.2)	135 (60.8)	222 (17.8)	
Multifocality				
Single	423 (46.3)	490 (53.7)	913 (73.3)	< 0.001*
Bifocal	43 (37.4)	62 (62.6)	115 (9.2)	
Multifocal	60 (27.5)	158 (72.5)	218 (17.5)	
Tumor size				< 0.001*
T1 (≤ 2 cm)	189 (57.6)	139 (42.4)	328 (26.3)	
T2 (2–5 cm)	319 (38.8)	504 (61.2)	823 (66.1)	
T3 and T4	18 (18.9)	77 (81.1)	95 (7.6)	
ER				
Negative	106 (47.3)	118 (52.7)	224 (18.0)	0.088
Positive	420 (41.1)	602 (58.9)	1022 (82.0)	
PR				
Negative	131 (41.5)	185 (58.5)	316 (25.4)	0.752
Positive	395 (42.5)	535 (57.5)	930 (74.6)	
HER-2				
Negative	446 (43.1)	590 (56.9)	1036 (83.1)	0.185
Positive	80 (38.1)	130 (61.9)	210 (16.9)	
Molecular subtype				0.031*
HR+/HER2−	372 (41.5)	524 (58.5)	896 (71.9)	
HR+/HER2+	50 (39.1)	78 (60.9)	128 (10.3)	
Enriched	30 (36.1)	53 (63.9)	83 (6.7)	
Tripple negative	74 (53.2)	65 (46.8)	139 (11.2)	
Ki-67 proliferation index				
Low (< 14)	66 (49.3)	68 (50.7)	134 (10.8)	0.198
Intermediate (14–19)	18 (41.9)	25 (58.1)	43 (3.5)	
High (≥ 20)	191 (40.6)	280 (59.4)	471 (37.8)	
Skin involvement				0.002*
Negative	500 (43.4)	651 (56.6)	1151 (92.4)	
Positive	26 (27.4)	69 (72.6)	95 (7.6)	
Nipple involvement				0.001*
Negative	507 (43.5)	659 (56.5)	1166 (93.6)	
Positive	19 (23.8)	61 (76.3)	80 (6.4)	
Axillary sonography				< 0.001*
Reactive LN	459 (79.3)	120 (20.7)	579 (46.5)	
LN with pathological features	67 (10.0)	600 (90.0)	667 (53.5)	
LVI				< 0.001*
Negative	340 (65.8)	177 (34.2)	517 (41.5)	
Positive	186 (25.5)	543 (74.5)	729 (58.5)	

*UOQ* upper outer quadrant, *LOQ* lower outer quadrant, *UIQ* upper inner quadrant, *LIQ* lower inner quadrant, *IDC* invasive ductal carcinoma, *ILC* invasive lobular carcinoma, *ER* estrogen receptor, *PR* progesterone receptor, *HER2* human epidermal growth factor receptor 2, *LVI* lympho-vascular invasion.

### Predictors of axillary lymph node metastasis

**Fig. 2:**
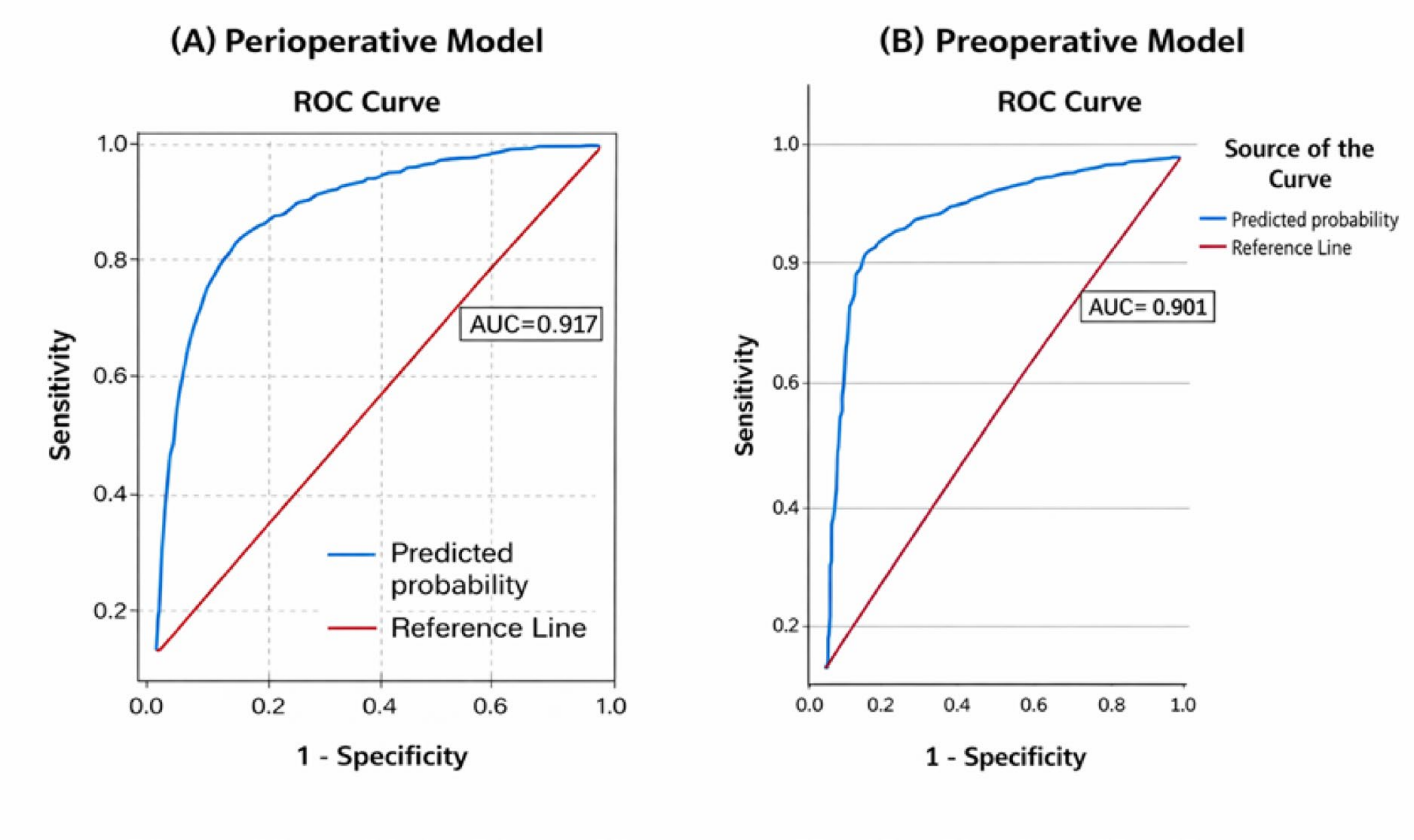
*Model discrimination performance.* Receiver operating characteristic (ROC) curves demonstrating the discriminative performance of the prediction models: (**A**) Perioperative Model (AUC = 0.917) and (**B**) Preoperative Model (AUC = 0.901). Both models show strong discrimination ability, as indicated by their respective AUC values.

Univariate logistic regression identified several variables that were significantly associated with axillary lymph node metastasis (Table 3). In the multivariate model, tumor location in the axillary tail and involvement of overlapping or multiple quadrants remained independent predictors, showing higher odds of nodal metastasis than centrally located tumors. Increasing tumor size was also independently associated, with T2 and T3/T4 tumors exhibiting progressively higher odds than T1 lesions. Triple-negative breast cancers demonstrated significantly lower odds of nodal involvement than luminal A tumors. Lymphovascular invasion was among the strongest predictors, conferring a more than threefold increased likelihood of ALN positivity. Preoperative Axillary ultrasonography was the most powerful predictor, with lymph nodes showing pathological features exhibiting markedly higher odds of metastasis than reactive nodes. The model demonstrated a good fit (Hosmer–Lemeshow *P* = 0.722) and explained a substantial proportion of the variance (Cox and Snell R^2^ = 0.477; Nagelkerke R^2^ = 0.641). Discrimination was excellent, with an ROC AUC of 0.917, supporting the robust prediction of axillary lymph node metastasis (Fig. 2).

**Table 3:** Risk factors for ALN identified by univariate and multivariate logistic regression.

Variables	Univariate analysis		Multivariate analysis	
	OR (95% CI)	*P* value	OR (95% CI)	*P* value
Quadrant		**< 0.001**		
Central region	Reference		Reference	
Axillary tail	6.999 (4.192–11.685)	**< 0.001**	4.648 (2.248–9.611)	**< 0.001**
Multiple sites	3.310 (1.852–5.918)	**< 0.001**	2.629 (1.118–6.183)	**0.027**
UOQ	1.495 (1.089–2.054)	**< 0.013**	1.785 (1.121–2.842)	**0.015**
LOQ	0.980 (0.573–1.674)	0.940	0.701 (0.314–1.568)	0.388
UIQ	0.491 (0.294–0.820)	**0.007**	0.771 (0.369–1.613)	0.490
LIQ	0.862 (0.521–1.425)	0.563	1.532 (0.748–3.141)	0.244
Histological grade		**0.038**		0.422
1	Reference		Reference	
2	2.255 (1.147–4.435)	**0.018**	0.734 (0.277–1.947)	0.534
3	2.549 (1.245–5.221)	**0.011**	0.981 (0.342–2.809)	0.971
Multifocality		**< 0.001**		0.621
Single	Reference		Reference	
Bifocal	1.445 (0.969–2.155)	0.071	1.122 (0.624–2.019)	0.700
Multifocal	2.273 (1.643–3.144)	**< 0.001**	1.278 (0.769–2.123)	0.343
Tumor size		**< 0.001**		**0.002**
T1 (≤ 2 cm)	Reference		Reference	
T2 (2 – 5 cm)	2.148 (1.656– 2.786)	**< 0.001**	1.594 (1.088–2.334)	**0.017**
T3 & T4	5.817 (3.329–10.162)	**< 0.001**	3.936 (1.745–8.879)	**0.001**
Molecular subtype		**0.033**		**< 0.001**
HR+/HER2-	Reference		Reference	
HR+/HER2+	1.107 (0.758–1.618)	0.598	0.847 (0.484–1.483)	0.562
HR^−^/HER2^+^	1.254 (0.786–2.001)	0.342	0.668 (0.314–1.420)	0.295
TNBC	0.624 (0.436–0.893)	**0.01**	0.273 (0.154–0.484)	**< 0.001**
LVI				
Negative	Reference		Reference	
Positive	5.608 (4.383–7.175)	**< 0.001**	3.836 (2.713–5.423)	**< 0.001**
Skin involvement				
Negative	Reference		Reference	
Positive	2.038 (1.279–3.248)	**0.003**	0.954 (0.450–2.021)	0.902
Nipple involvement				
Negative	Reference		Reference	
Positive	2.470 (1.457–4.187)	**0.001**	1.686 (0.772–3.682)	0.190
Axillary sonography				
Reactive LN	Reference		Reference	
Pathological Features	34.254 (24.80–47.298)	**< 0.001**	27.740 (19.269–39.936)	**< 0.001**

Hosmer and Lemeshow Test (P = 0.722), Cox and Shell R Square (0.477), Nagelkerke R Square (0.641).

### Construction and validation of the nomogram

**Fig. 3:**
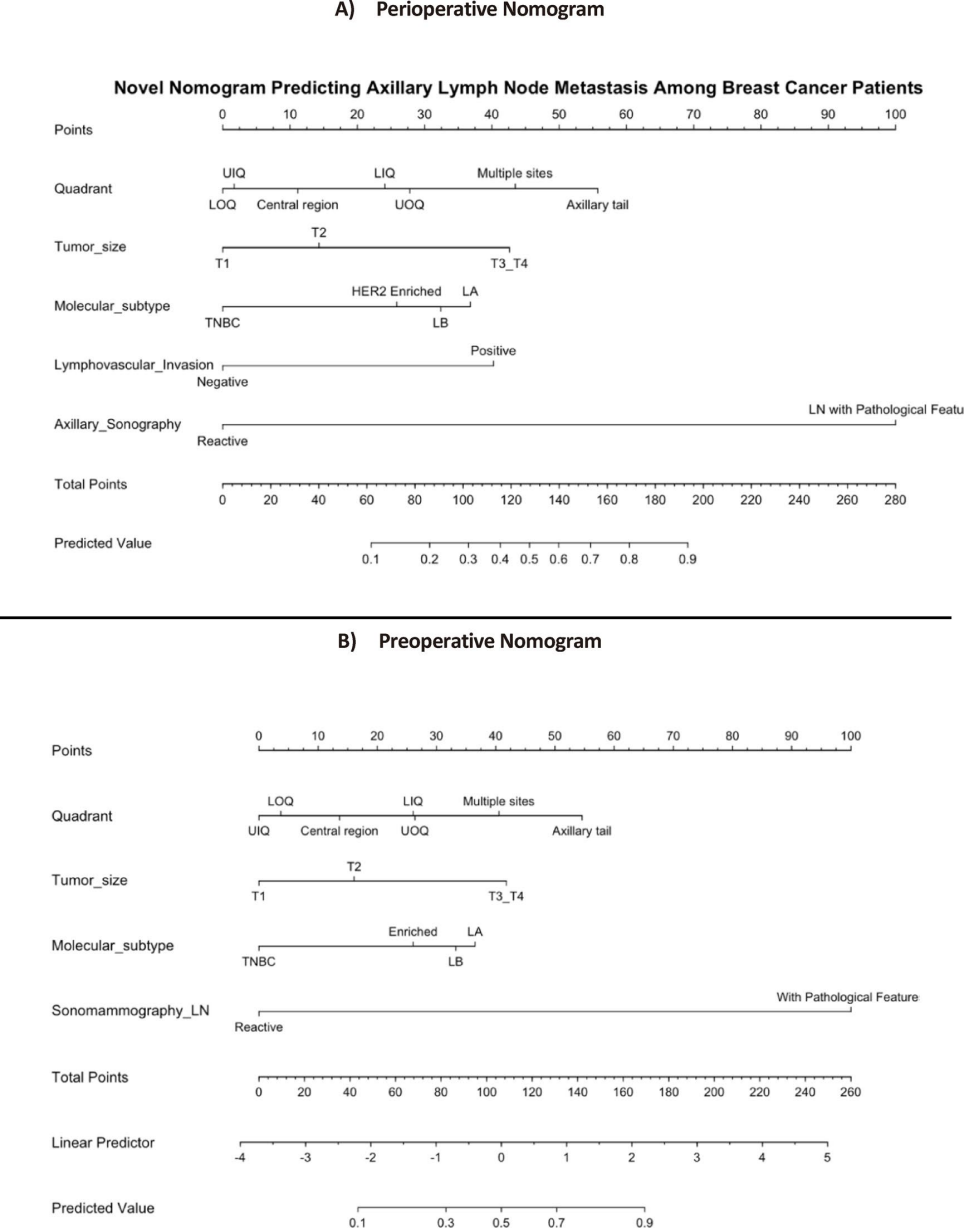
(**A**) Nomogram for predicting the probability of axillary lymph node (ALN) metastasis using perioperative variables. Scores for each of the five predictors (rows 2–6) are mapped to the “Points” row (row 1), summed in the “Total Points” row (row 7), and translated into the predicted risk of ALN metastasis in row 8. (**B**) Nomogram for predicting the probability of axillary lymph node (ALN) metastasis in the preoperative-only sensitivity model excluding LVI.

A nomogram was constructed based on five independent predictors identified in the multivariate logistic regression analysis: tumor quadrant location, tumor size, molecular subtype, lymphovascular invasion, and axillary ultrasonography findings (Fig. 3). Each variable was assigned a weighted score that reflected its relative contribution to axillary lymph node metastasis. Axillary ultrasonography with pathological lymph node features showed the greatest weight (maximum, 100 points), followed by tumor quadrant location (up to 60 points), with higher scores for tumors in the axillary tail or multiple quadrants. Tumor size was scored proportionally, with larger tumors (T2–T3/T4) receiving higher scores than T1 lesions. Lymphovascular invasion contributed moderately, while the molecular subtype had the least weight (up to 38 points), with triple-negative tumors receiving lower scores owing to lower odds of nodal involvement. The total score across all variables corresponded to the predicted probability of axillary lymph node metastasis, enabling individualized risk assessment.

**Fig. 4:**
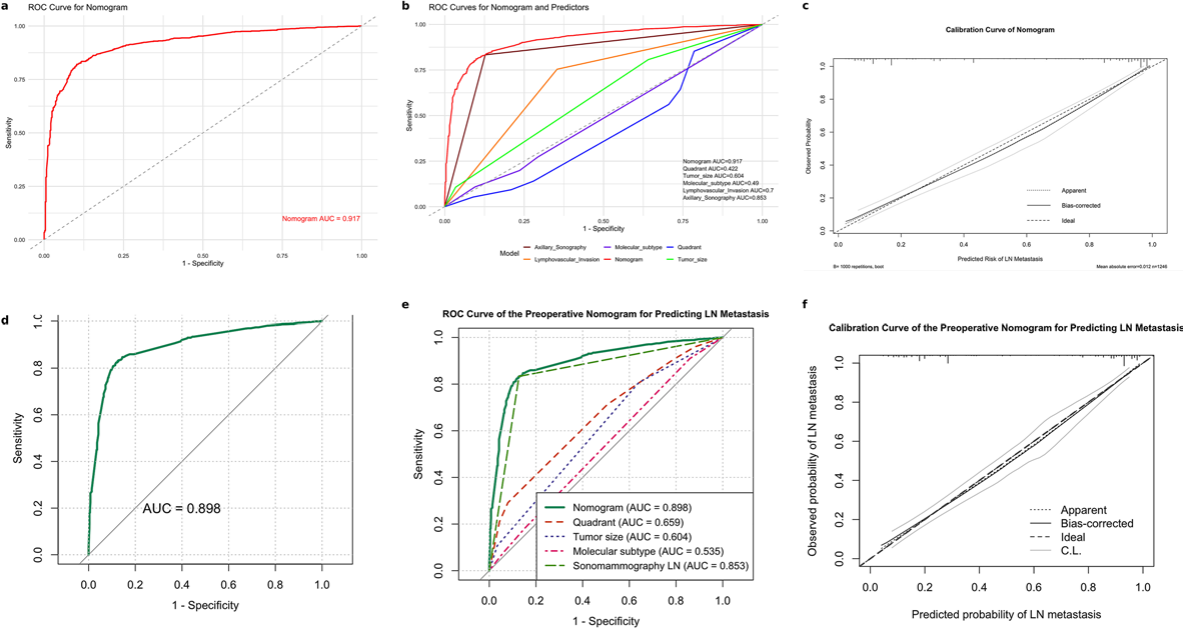
*Nomogram performance for predicting axillary lymph node metastasis:* Panels (**a**–**c**) present the performance of the perioperative model, and panels (**d**–**f**) present the performance of the preoperative model. (**a**) ROC curve of the perioperative nomogram (AUC = 0.917). (**b**) ROC curves for the nomogram versus individual predictors: quadrant (0.422), tumor size (0.604), molecular subtype (0.490), lympho-vascular invasion (0.700), and axillary sonography (0.853). (**c**) Calibration curve of the perioperative model showing agreement between predicted and observed probabilities; curves closer to the ideal reference line indicate better calibration. (**d**) ROC curve of the preoperative nomogram (AUC = 0.898). (**e**) ROC curves comparing the preoperative nomogram with individual predictors: quadrant (AUC = 0.659), tumor size (AUC = 0.604), molecular subtype (AUC = 0.535), and sonomammography lymph node assessment (AUC = 0.853). (**f**) Calibration curve of the preoperative model demonstrating good concordance between predicted and observed probabilities.

The nomogram demonstrated excellent predictive performance with a concordance index (C-index) of 0.917, indicating a strong discriminative ability for axillary lymph node metastasis. This was supported by the ROC curve, which confirmed the high accuracy of the model (Fig. 4a,b). The calibration curve further showed good agreement between the predicted and observed probabilities, indicating the reliable performance of the nomogram in this cohort (Fig. 4c).

The apparent C-index of the model was 0.917. After optimism correction using bootstrap resampling (1,000 iterations), the model maintained excellent discrimination, with a corrected C-index of 0.902 (95% CI, 0.889–0.915). Calibration assessment showed a calibration slope of 0.94 and an intercept of 0.03, with a mean absolute error of 0.021 between predicted and observed probabilities, indicating good overall model calibration.

Model stability was further supported by tenfold cross-validation, which yielded a mean AUC of 0.908 (range, 0.892–0.921), consistent with the bootstrap-corrected estimates.

The diagnostic performance of the nomogram across clinically relevant risk thresholds (10%, 20%, and 30%) is summarized in (Table 4). At the 10% threshold, the model demonstrated a very high sensitivity (98.5%), ensuring that nearly all patients with nodal metastasis were correctly identified; however, the accompanying low specificity (26.0%) limits its utility as a practical triage cutoff. At the 20% threshold, our recommended clinical decision point, the nomogram achieved a favorable balance with 93.9% sensitivity and 59.5% specificity, yielding an NPV of 87.7%. In practical terms, patients classified as low-risk (< 20% predicted probability) would have only a 12.3% likelihood of missing nodal disease, supporting the safe omission of axillary surgery in a substantial proportion of cases while maintaining an acceptably low miss rate. At the 30% threshold, the sensitivity remained high (92.4%) with improved specificity (66.9%) and NPV (86.5%), offering a viable alternative cutoff for settings where clinicians prioritize greater selectivity in omitting axillary procedures while preserving strong reassurance against missed metastasis.

**Table 4:** Diagnostic accuracy of the nomogram at clinically relevant thresholds.

Risk threshold	Sensitivity (%)	Specificity (%)	PPV (%)	NPV (%)	Accuracy (%)
0.1	98.5	26.0	64.6	92.6	67.9
0.2	93.9	59.5	76.0	87.7	79.4
0.3	92.4	66.9	79.3	86.5	81.6

Performance metrics expressed as percentages.

In verification-bias sensitivity analyses, the model was refitted separately within the SLNB and ALND subgroups. Discriminative performance remained high in both groups (SLNB AUC = 0.875; ALND AUC = 0.908). The effect estimates and performance of the ALND-only model were highly consistent with the primary combined-cohort model, indicating that the results were not materially driven by differences in nodal verification method (Supplementary File S1).

**Fig. 5:**
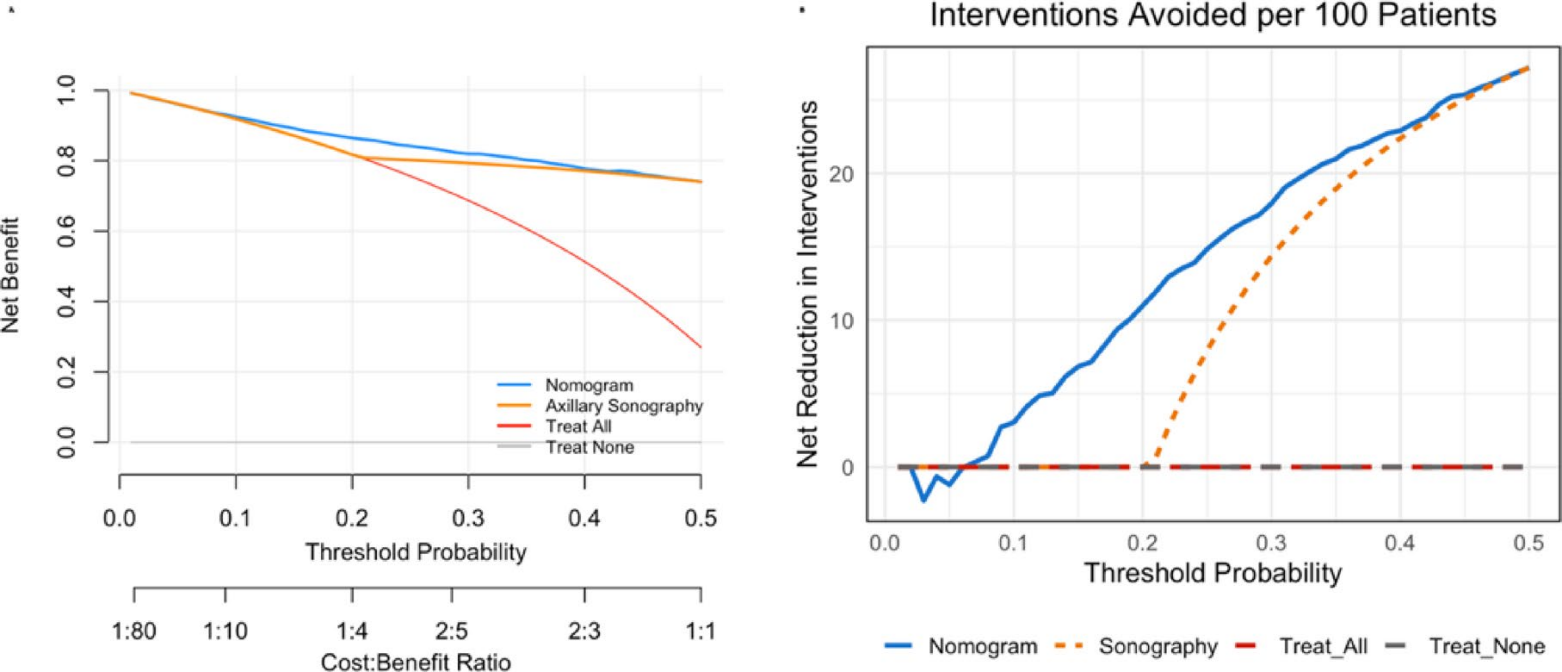
(**A**) Decision curve analysis comparing the nomogram and axillary sonography with the “treat all” and “treat none” strategies across threshold probabilities. (**B**) Net reduction in interventions per 100 patients, illustrating the clinical utility of the nomogram and axillary sonography at varying threshold probabilities.

Decision curve analysis demonstrated that the nomogram provided a consistently higher net benefit than either axillary sonography alone or the default “treat all” and “treat none” strategies across clinically relevant threshold probabilities (Fig. 5A). At low thresholds (around10%), the nomogram and sonography performed similarly; however, once the threshold exceeded approximately 20%, the nomogram clearly outperformed sonography, offering superior risk discrimination and greater clinical net benefit.

In the net reduction in the intervention plot (Fig. 5B), the nomogram also resulted in a greater reduction in unnecessary axillary dissections than axillary sonography, particularly within the clinically relevant threshold range. At a 20% threshold probability, the nomogram avoided approximately 11 unnecessary dissections per 100 patients, whereas sonography alone did not reduce overtreatment. This advantage increased at higher thresholds, with the nomogram avoiding up to 27.2 unnecessary dissections per 100 patients at a 50% threshold, reflecting a meaningful reduction in overtreatment within the clinically relevant decision range.

### Preoperative-only model (excluding LVI)

To assess the robustness of the primary model, we performed a sensitivity analysis by refitting a preoperative-only model that excluded LVI. This alternative model demonstrated slightly lower discrimination than the primary perioperative model, but maintained acceptable predictive performance (C-index = 0.898; AUC = 0.898 vs. 0.917 in the primary model). The calibration remained acceptable, and the direction and magnitude of the regression coefficients were generally consistent with those of the primary model. These results are presented alongside the primary perioperative model in (Figs. 2, 3, and 4), allowing direct comparison of model discrimination, nomogram, and performance metrics.

## Discussion

The management of BC has progressively evolved toward precision and individualized care, with a growing emphasis on achieving therapeutic benefits while minimizing treatment-related morbidity. Although SLNB and ALND are routinely used in clinical practice to determine axillary lymph node status, both are invasive and are accompanied by varying degrees of postoperative complications. Consequently, modern surgical oncology increasingly favors selective interventions over routine axillary interventions^17,18^.

The relatively high proportion of ALND in this cohort reflects the combined influence of the underlying disease burden and resource-related constraints rather than deviation from guideline-directed care. Many patients presented with clinically node-positive or higher-stage disease, and during part of the study period, the dual-tracer SLNB technique was not universally available; therefore, ALND was performed when SLNB could not be performed.

Landmark randomized trials over the past decade have reshaped axillary management of BC, including ACOSOG Z0011, AMAROS trials, and IBCSG 23–01, collectively support global movement toward morbidity-sparing axillary de-escalation^9,19,20^. However, translating these evidence-based practices into routine clinical care remains challenging in some resource-constrained settings, where consistent adoption of SLNB is limited, and surgeons may still default to ALND for staging and treatment^21^.

Our study developed and validated a highly accurate nomogram for the preoperative prediction of ALN metastasis among BC patients in Egypt, achieving an impressive C-index of 0.917. Several nomograms have been developed internationally, most of which focus on calculating the risk of sentinel axillary node^22,23^ or additional non-sentinel node involvement^24,25^, limiting their utility in clinical contexts where SLNB may not always be feasible. Few existing tools aim to predict overall axillary node metastasis, and even fewer have been adapted to non-Western populations.

Our model identified five key predictors of ALN metastasis: tumor location, tumor size, lymphovascular invasion, molecular subtype, and pathological features on axillary ultrasound, which were consistently aligned with established patterns of metastatic behavior. The diagnostic performance of the nomogram (AUC 0.917) exceeded that of axillary ultrasonography alone (AUC 0.853) and far outperformed individual predictors, such as tumor size (AUC 0.604) and molecular subtype (AUC 0.490). Notably, its discriminative performance compares favorably with previously published models, including those by Lei et al., Zong et al., and Tang et al., which reported AUC values of 0.765, 0.802, and 0.815, respectively^26–28^. Although ultrasound is widely used for axillary evaluation, its reported false-negative rates of up to 22.9% limit its reliability as a standalone modality^11^.

Although our model achieved high discrimination (AUC 0.917), this performance should be interpreted in the context of our clinical setting. Our institution is a tertiary referral center with a high proportion of patients presenting with advanced disease and a relatively high prevalence of nodal metastasis. In addition, the strong predictive contribution of axillary ultrasonography in our model likely reflects both the quality of imaging assessment at our center and the greater burden of nodal disease in this population, rather than overfitting or artificial model inflation.

We acknowledge that although the model was developed to guide preoperative decision support, LVI was available from both biopsy and postoperative pathology reports in our cohort, and postoperative histopathology represents a more reliable source of LVI assessment in many cases. This mixed availability limits the extent to which the primary model can be considered strictly preoperative. To address this limitation, we refitted a preoperative-only model excluding LVI, which retained excellent discrimination and performance that was highly comparable to that of the primary model (C-index/AUC = 0.898). These findings indicate that the model remains robust when restricted to preoperative information, thereby supporting its potential applicability for preoperative clinical risk stratification.

Our results showed that tumors arising in the axillary tail or involving multiple or overlapping quadrants had markedly higher metastatic potential, consistent with their closer drainage to level I axillary nodes, a relationship infrequently highlighted in Western prediction models^29–31^. The nearly five-fold increased risk associated with axillary-tail tumors carries meaningful surgical implications in settings where SLNB may be unavailable, as these patients may be more appropriately directed toward upfront ALND when selective staging is not feasible. In our cohort, UOQ tumors also showed significantly increased odds of nodal involvement (OR = 1.785, P = 0.015), aligning with prior studies reporting a higher prevalence of nodal metastasis in outer-quadrant tumors than in inner-quadrant lesions^32,33^.

In our cohort, HER2-positive tumors were associated with more lymph node positivity than HER2-negative tumors in the primary cohort (61.9% vs. 56.9%), while triple-negative tumors showed a lower risk of ALN metastasis than Luminal A disease. These findings align with those of prior studies showing that triple-negative tumors have the lowest likelihood of nodal metastasis, whereas HER2-enriched tumors have the highest propensity for nodal metastasis^34,35^. The protective effect of TNBC in our model was particularly notable, with an odds ratio of 0.273 (95% CI 0.154–0.484, *p* < 0.001), suggesting distinct biological behavior in our population that deserves further investigation.

Our model also reflects the clinical reality of breast cancer presentation in Egypt, where a substantial proportion of patients present with T2 or larger tumors and locally advanced features^36^. This late-stage pattern elevates the baseline probability of nodal metastasis and limits the applicability of Western-developed nomograms built predominantly from early stage cohorts. By incorporating variables such as tumor size, skin or nipple involvement, and axillary ultrasound characteristics, which are features more common in our population, the model achieves greater contextual relevance for local practice. These considerations are particularly important in settings where SLNB pathways may be inconsistently available and where avoiding unnecessary ALND can reduce morbidity, conserve resources, and minimize the burden of repeat surgery for patients who often face significant travel barriers.

Decision curve analysis demonstrated that the nomogram provides a consistently greater net benefit across clinically relevant thresholds, particularly above 20%, where it avoids approximately 11 unnecessary dissections per 100 patients compared to ultrasound alone. This clinical utility is especially valuable in resource-limited settings, where avoiding overtreatment is essential for optimizing care efficiency and reducing morbidity.

Based on our results, these threshold-based scenarios should be regarded as exploratory and hypothesis-generating rather than prescriptive recommendations; however, a potential clinical implementation pathway may be considered: patients with a predicted risk below 10% may represent a very low-risk subgroup in whom routine ALND could potentially be reconsidered, given the model’s 98.5% sensitivity at this threshold ; for example, in a clinically node-negative patient with negative axillary ultrasonography and a predicted ALNM risk < 10%, the model may support multidisciplinary team discussion regarding the omission of ALND in settings where SLNB is unavailable; those with intermediate risk (10–30%) could be evaluated further with adjunct imaging or SLNB when available; and patients exceeding a 30% predicted risk may warrant consideration for more aggressive axillary treatment, effectively adapting Western de-escalation principles to local realities in a research-guided context.

At the 20% threshold, the nomogram achieved a favorable balance with 93.9% sensitivity and 59.5% specificity, yielding an NPV of 87.7%. In practical terms, patients classified as low-risk (< 20% predicted probability) would have only a 12.3% likelihood of missing nodal disease, which may support selective evaluation of omission strategies in carefully controlled clinical pathways. Such a framework aligns with global movements toward the de-escalation of axillary surgery, as explored in trials such as SOUND, while the complexity highlighted in SENTINA underscores the need for cautious context-specific validation. Accordingly, any modification of axillary surgery based on model-derived thresholds should be confirmed through prospective external validation and outcome-based studies, particularly in low- and middle-income countries (LMICs)^37,38^.

External validation and generalizability. Although the model demonstrated excellent discrimination and calibration following internal optimization correction, internal validation could not fully account for performance optimism or guarantee reproducibility in different practical environments. Our cohort was derived from a setting with a relatively high prevalence of ALNM, which may partly contribute to the high discrimination observed and may not reflect populations presenting with earlier-stage disease or broader SLNB adoption. Accordingly, external validation in independent cohorts is essential before clinical implementation. We also indicate that prospective multicenter external validation is currently being initiated through collaboration with other regional cancer centers.

## Conclusion

The current study has the potential to improve BC care in LMICs by addressing critical gaps in evidence-based axillary management. By leveraging nationally generated data from a major tertiary center in Egypt, it provided population-specific evidence that is directly relevant to local clinical practice. The model was evaluated for its applicability, performance, and potential integration into existing care pathways, demonstrating its strong accuracy and practical value in settings where axillary staging resources may be inconsistent. Ultimately, this work contributes to advancing context-appropriate decision support for axillary surgery and offers a framework for incorporating evidence-based tools into healthcare systems across similar resource-constrained environments.

## Limitations

This single-center, retrospective design limits formal external validation, an important step for confirming model generalizability; therefore, model performance should be interpreted with caution outside similar clinical settings. However, within the Egyptian healthcare context, which is characterized by a centralized oncology system, standardized diagnostic pathways, and relatively homogeneous patterns of BC presentation, our cohort likely reflects the broader national population. External validation through multicenter collaboration would strengthen the model further, but such efforts are currently challenged by fragmented health records and limited inter-institutional data sharing, which are issues common across many LMICs. To address this issue, collaborative partnerships with regional cancer centers are being initiated to enable future prospective external validation. Until such validation is achievable, our internally validated nomogram provides a robust and contextually relevant tool to support more selective axillary management in resource-constrained environments.

## Supplementary Information

Supplementary Information.

## Data Availability

The datasets generated and/or analyzed during the current study are available from the corresponding author upon reasonable request.
