# Supplementary material for: Development and validation of a novel nomogram predicting axillary lymph node metastasis among breast cancer patients in Egypt
Source: Sci Rep. 2026 Feb 17;16:7187. doi: 10.1038/s41598-026-37354-9 (PMC12921286; doi:10.1038/s41598-026-37354-9)
Supplement: Supplementary file 1 — Supplementary Information. [file 41598_2026_37354_MOESM1_ESM.docx]

**Supplementary File S1. Verification-bias sensitivity modelling in SLNB and ALND patient subgroups**

**Table S1. Multivariable logistic regression results from subgroup prediction models in SLNB and ALND cohorts (sensitivity analysis)**

| **Variables** | **SLNB Cohort**  **(N= 277)** |  | **ALND Cohort**  **(N=969)** | |  |
| --- | --- | --- | --- | --- | --- |
|  | |  |  |  |  |
|  | **OR (95% CI)** | **P value** | **OR (95% CI)** | **P value** |  |
| **Quadrant** |  | 0.118 |  | **0.002** |  |
| Central region | Reference |  | Reference |  |  |
| Axillary tail | 3.114 (0.457 – 21.211) | 0.246 | 5.724 (2.388 – 13.274) | **<0.001** |  |
| Multiple sites | | 3.900 (0.557 – 27.306) | 0.170 | 1.662 (0.633 – 4.365) | 0.303 |
| UOQ | | 0.867 (0.278 – 2.710) | 0.806 | 1.759 (1.049 – 2.949) | **0.032** |
| LOQ | 0.966 (0.137 – 6.792) | 0.972 | 0.652 (0.263 – 1.615) | 0.355 |  |
| UIQ | 0.104 (0.010 – 1.092) | 0.059 | 1.251 (0.520 – 3.009) | 0.618 |  |
| LIQ | 2.463 (0.571 – 10.622) | 0.227 | 1.558 (0.652 – 3.719) | 0.318 |  |
| **Histological Grade** |  | **0.036** |  | 0.941 |  |
| 1 | Reference |  | Reference |  |  |
| 2 | 0.773 (0.071–8.357) | 0.832 | 1.201 (0.413 – 3.489) | 0.737 |  |
| 3 | 3.623 (0.294–44.593) | 0.315 | 1.225 (0.385 – 3.898) | 0.732 |  |
| **Multifocality** |  | 0.189 |  | 0.587 |  |
| Single | Reference |  | Reference |  |  |
| Bifocal | 0.643 (0.151 – 2.745) | 0.551 | 1.295 (0.620 – 2.708) | 0.491 |  |
| Multifocal | 2.622 (0.805 – 8.541) | 0.110 | 1.279 (0.734 – 2.229) | 0.384 |  |
| **Tumor size** |  | 0.500 |  | **0.011** |  |
| T1 ( ≤ 2cm) | Reference |  | Reference |  |  |
| T2 (2 – 5 cm) | 1.439 (0.584– 3.548) | 0.429 | 1.581 (1.001 – 2.497) | **0.050** |  |
| T3 & T4 | 3.489 (0.324 – 37.593) | 0.303 | 3.542 (1.487 – 8.436) | **0.004** |  |
| **Molecular Subtype** |  | **0.014** |  | **<0.003** |  |
| HR+/HER2- | Reference |  | Reference |  |  |
| HR+/HER2+ | 0.606 (0.050 – 7.292) | 0.693 | 0.603 (0.276 – 1.315) | 0.203 |  |
| HR^−^/HER2^+^ | 0.149 (0.020 – 1.102) | 0.062 | 1.008 (0.533 – 1.907) | 0.979 |  |
| TNBC | 0.088 (0.017 – 0.446) | **0.003** | 0.300 (0.156 – 0.577) | **<0.001** |  |
| **Skin Involvement** |  |  |  |  |  |
| Negative | Reference |  | Reference |  |  |
| Positive | - |  | 1.199 (0.533 – 2.693) | 0.661 |  |
| **Nipple Involvement** | |  |  |  |  |
| Negative | Reference |  | Reference |  |  |
| Positive | - |  | 1.686 (0.714 – 3.983) | 0.234 |  |
| **Axillary Sonography** |  |  |  |  |  |
| Reactive LN | Reference |  | Reference |  |  |
| Pathological Features | 24.833(9.450 – 65.254) | **<0.001** | 32.060 (20.904 – 49.170) | **<0.001** |  |


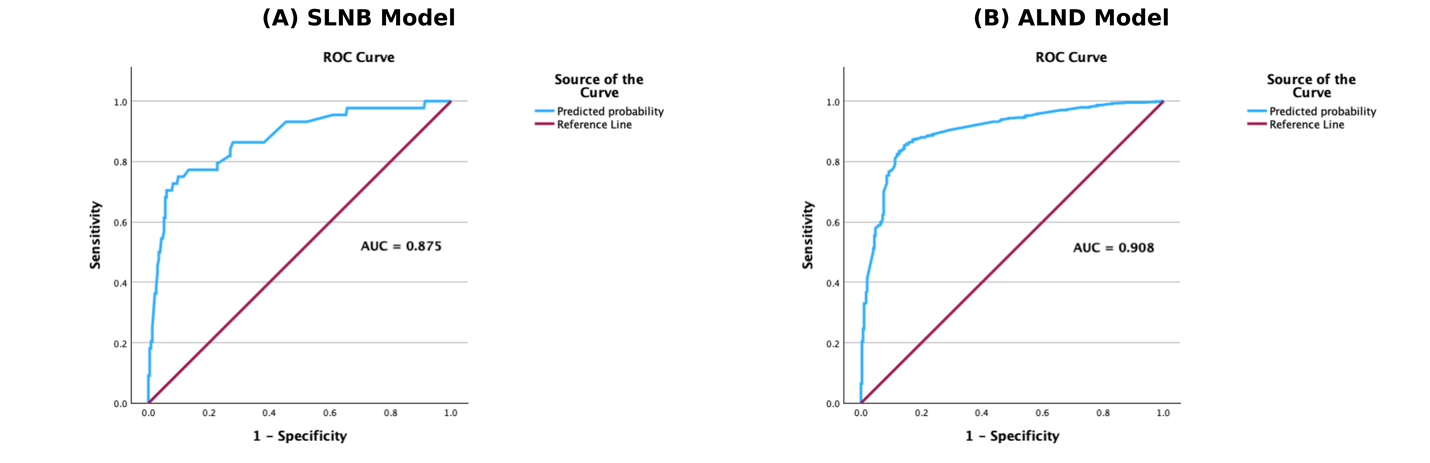


**Figure S1. *Discriminative performance of the SLNB and ALND prediction models.***(A) Receiver operating characteristic (ROC) curve of the SLNB model demonstrating good discrimination (AUC = 0.875). (B) ROC curve of the ALND model demonstrating strong discrimination (AUC = 0.908)
